# Targeting c‐met receptor tyrosine kinase by the DNA aptamer SL1 as a potential novel therapeutic option for myeloma

**DOI:** 10.1111/jcmm.13870

**Published:** 2018-10-24

**Authors:** Yibin Zhang, Hongmei Gao, Weihua Zhou, Sunming Sun, Yayue Zeng, Hui Zhang, Long Liang, Xiaojuan Xiao, Jianhui Song, Mao Ye, Yujia Yang, Jingfeng Zhao, Zi Wang, Jing Liu

**Affiliations:** ^1^ Key Laboratory of Nanobiological Technology of Chinese Ministry of Health Xiangya Hospital Central South University Changsha China; ^2^ Molecular Biology Research Center & Center for Medical Genetics School of Life Sciences Central South University Changsha China; ^3^ Molecular Science and Biomedicine Laboratory State Key Laboratory for Chemo/Biosensing and Chemometrics College of Biology College of Chemistry and Chemical Engineering Hunan University Changsha China; ^4^ Nursing Department Xiangya Hospital Central South University Changsha China; ^5^ Department of Pediatrics Xiangya Hospital Central South University Changsha China

**Keywords:** aptamer, bortezomib, CLN0003_SL1, c‐met receptor, multiple myeloma

## Abstract

Hepatocyte growth factor (HGF)/c‐met pathway activation has been implicated in the pathogenesis of multiple myeloma (MM), and blocking this pathway has been considered a rational therapeutic strategy for treating MM. Aptamers are single‐stranded nucleic acid molecules that fold into complex 3D structures and bind to a variety of targets. Recently, it was reported that DNA aptamer SL1 exhibited high specificity and affinity for c‐met and inhibited HGF/c‐met signaling in SNU‐5 cells. However, as the first c‐met‐targeted DNA aptamer to be identified, application of SL1 to myeloma treatment requires further investigation. Here, we explore the potential application of SL1 in MM. Our results indicated that c‐met expression is gradually increased in MM patients and contributes to poor outcomes. SL1 selectively bound to c‐met‐positive MM cells but not to normal B cells and suppressed the growth, migration and adhesion of MM cells in vitro in a co‐culture model performed with HS5 cells, wherein SL1 inhibited HGF‐induced activation of c‐met signaling. In vivo and ex vivo fluorescence imaging showed that SL1 accumulated in the c‐met positive tumour areas. In addition, SL1 was active against CD138+ primary MM cells and displayed a synergistic inhibition effect with bortezomib. Collectively, our data suggested that SL1 could be beneficial as a c‐met targeted antagonist in MM.

## 1 INTRODUCTION

Multiple myeloma (MM) is a B‐cell malignancy that is characterized by the monoclonal expansion of abnormal plasma cells (PC) in the bone marrow (BM), which leads to devastating clinical manifestations such as hypercalcemia, osteolytic lesions, anaemia, and impaired renal function. MM accounts for 15%‐20% of deaths related to hematological malignancy, or 2% of all cancer deaths, and primarily affects elderly individuals with a median age at diagnosis of 69 years.^1^ While the introduction over the last decade of high‐dose chemotherapy, autologous stem cell transplantation, proteasome inhibitors, immunomodulatory agents, and their combinations has prolonged the survival of patients with newly diagnosed MM, patients ultimately relapse, and those with relapsed/refractory MM often respond poorly to standard agents^2^, ^3^; thus, new treatment options are urgently needed.

Most of MM development depends on the interactions between MM cells and components of the bone marrow microenvironment (BMME) and the signaling that results.^4^ One promising candidate signaling pathway is the hepatocyte growth factor (HGF)/c‐met pathway. HGF is a constituent of the BMME and the specific ligand for the tyrosine kinase receptor c‐met.^5^ Upon HGF binding, c‐met dimerizes, resulting in trans‐phosphorylation of two tyrosine residues (Y1234 and Y1235) in the kinase domain followed by auto‐phosphorylation of two tyrosine residues (Y1349 and Y1356) in the C‐terminal region. Phosphorylation of Y1349 and Y1356 creates a multisubstrate docking site that is necessary for the induction of downstream signaling cascades such as Ras/Raf/MAPK, PI3K/AKT/mTOR, and/or STAT3/5. These signaling cascades drive distinct biological responses including cell growth, inhibition of apoptosis, cell migration and angiogenesis.^6^ MM cells express c‐met and often simultaneously express HGF, thus creating an HGF/c‐met autocrine loop. HGF is also secreted by BM stromal cells, which provides an additional paracrine loop to stimulate tumour‐stromal crosstalk that favours MM cells growth and metastasis.^7^ Clinically, c‐met is not mutated in MM, but MM patients have high serum levels of HGF and high *c‐met* expression and gene copy number, which are correlated with poor prognosis and advanced disease.^8^, ^9^, ^10^, ^11^ It has been demonstrated that abnormal activation of the HGF/c‐met pathway supports MM cell survival, growth, angiogenesis, osteolytic lesions and drug resistance.^5^, ^6^ Thus, the HGF/c‐met interaction has recently emerged as a promising target in MM therapy.

Recently, several antibodies/agents that interfere with HGF/c‐met signaling have entered preclinical or clinical trials including ligand antagonists (monoclonal antibody),^12^ receptor inhibitors (monoclonal antibody)^13^ and receptor kinase inhibitors.^6^ However, inherent limitations of these antibodies/inhibitors,^14^, ^15^ such as cellular cytotoxicity or off‐target effects, limit their clinical use and prompted the development of a new class of therapeutic antagonists, namely, aptamers. Aptamers are single‐stranded oligonucleotides that are isolated from RNA or ssDNA libraries via systematic evolution of ligands by exponential enrichment (SELEX).^16^ Similar to antibodies, aptamers bind to their targets with high affinity and selectivity due to their unique three‐dimensional structures. However, aptamers are advantageous over antibodies due to their low potential for immunogenicity, efficient tissue penetration, relatively simple synthesis, etc.^17^ To date, a small number of aptamers have been developed as therapeutic antagonists in MM,^18^, ^19^ but none target c‐met.

Recently, DNA aptamer CLN0003 (CLN3) was isolated from Jurkat cells via Cell‐ SELEX and was found to bind c‐met with high specificity and affinity.^20^ Ueki et al identified the 50‐mer minimal binding motif of CLN3 (SL1) that retained high c‐met affinity and interfered with HGF binding to c‐met in SNU‐5 cells.^21^ However, whether SL1 can become the first aptamer to target c‐met in MM requires further investigation. In this work, we characterized the clinical significance of *c‐met* in MM and studied the selectivity and binding properties of SL1 in MM via a series of in vitro, in vivo and ex vivo assays. Furthermore, we showed that SL1 has the potential for treating clinical MM cells that express CD138, a hallmark of malignant PC. Furthermore, we show that SL1 can be used in combination with the first‐line drug, bortezomib (BTZ). In all, our data support SL1 as a promising molecular tool for developing new MM treatments.

## 2 MATERIALS AND METHODS

### 2.1 Cell lines and cell culture

ARP‐1 and HS5 cell lines were obtained from the Institute of Hematology & Blood Diseases Hospital, Chinese Academy of Medical Science & Peking Union Medical College, Tianjin, China. MM.1S cell lines were obtained from the American Type Culture Collection (ATCC, USA). Human peripheral B lymphocytes (B‐cells) were obtained from the State Key Laboratory of Medical Genetics, Changsha, China. B cells, ARP‐1 and MM.1S cell lines were cultured in RPMI 1640 medium (Gibco, New York, NY, USA) supplemented with 10% foetal bovine serum (FBS; Gibco). HS5 cells were cultured in DMEM medium (HyClone, Logan, UT, USA) supplemented with 10% FBS. All cells were cultured in a humidified incubator at 37°C and 5% CO_2_.

### 2.2 Aptamers, reagents and antibodies

The ssDNA library used in this study contained a random sequence of 40 nucleotides flanked by a 5′ primer‐hybridizing sequence of 22 nucleotides and a 3′ primer‐hybridizing sequence of 24 nucleotides (5′‐GGAGGGAAAAGTTATCAGGC‐(N)40‐GATTAGTTTTGGAGTACTCGCTCC‐3′). The SL1 sequence was as follows: 5′‐ATCAGGCTGGATGGTAGCTCGGTCGGGGTGGGTGGGTTGGCAAGTCTGAT‐3′. All DNA sequences were synthesized and HPLC‐purified by Sangon Biotech Co. Ltd. (Shanghai, China). Recombinant human HGF (#100‐39) was obtained from Peprotech (Rocky Hill, NJ, USA). Tivantinib/ARQ197 (S2753) was purchased from Selleck Chemicals (Houston, TX, USA). Antibodies against c‐met (#8198), phosphorylated c‐met (#3133), and GAPDH (#5174) were purchased from Cell Signaling Technology (Boston, MA, USA). Antibodies against α‐tubulin (sc‐5286), p‐ERK (sc‐7383), Akt1 (sc‐5298), p‐Akt (sc‐16646‐R), and ERK1/2 (sc‐514302) were purchased from Santa Cruz (Santa Cruz, CA, USA). CD138 microbeads (130‐051‐301) were purchased from Miltenyi Biotec (Bergisch Gladbach, Germany).

### 2.3 Gene expression profile accession numbers

The gene expression profile (GEP) accession number for the microarrays performed on 44 subjects with MGUS, 22 healthy donors, and 559 newly diagnosed MM patients reported in this study to evaluate the expression of c‐met are GSE 5900 and GSE 2658.

### 2.4 Western blot analysis

As described previously,^22^ cells were lysed with RIPA buffer (Beyotime, Shanghai, China) that contained a protease and phosphatase inhibitor mixture (Roche, Mannheim, Germany) and cells membrane protein were extracted by membrane and cytosol protein extraction kit(P0033; Beyotime). Protein extraction (50 μg) were boiled and subjected to a 10% SDS‐PAGE gel followed by immunoblotting with specific antibodies.

### 2.5 Aptamer binding specificity and dissociation constant (*K*_*d*_) determination

Cells (3 × 10^5^) were incubated with varying concentrations of a FAM‐labelled control library or SL1 in 200 μL of binding buffer (1× PBS with 4.5 g/L glucose, 5 mM of MgCl_2_, 0.1 mg/mL yeast tRNA, and 1 mg/mL BSA) at 4°C for 30 minutes. Cells were washed three times with 0.5 mL of washing buffer (1× PBS with 4.5 g/L glucose and 5 mM of MgCl_2_) and then resuspended in 0.5 mL of PBS for flow cytometry analysis (BD FACSVerse™, BD Biosciences, New York, NY, USA). After subtracting the mean fluorescence intensity of non‐specific binding from the DNA library, the *K*_*d*_ of SL1 was determined by fitting the dependence of fluorescence intensity of specific binding on the concentration of SL1 to the equation: *Y* = *B* max *X*/(*K*_*d*_ + *X*) performed with GraphPad Prism 7.0 (GraphPad software, La Jolla, CA, USA).

### 2.6 Co‐culture system

HS5 BM stromal cells were first seeded into 96‐well plates (0.2 × 10^5^ cells/100 μL/well) or the bottom chamber of a Transwell system (4 × 10^5^ cells/1 mL/well) for at least 24 hours to permit cell adhesion and the formation of a confluent monolayer. The non‐adherent cell fraction was removed, and the adherent monolayer was washed with PBS. B cells or MM cells were then added to the prepared adherent stroma either directly (cell‐on‐cell) in plates or indirectly (separated by a micropore membrane) in the upper chamber of a Transwell system.

### 2.7 SiRNA transfection

Cells (3 × 10^5^ cells/well) were seeded in six‐well plates and transfected with 100 nM/well of human‐c‐met siRNA or scramble siRNA performed with the X‐tremeGENE siRNA transfection reagent, according to the manufacturer's instructions (Roche, Basel, Switzerland). Forty‐eight hours after transfection, the cells were used for further experiments. C‐met siRNA sequence: 5′‐CCAAUGGAUCGAUCUGCCATT‐3′; Scramble siRNA sequence: 5′‐ACUACCGUUGUUAUAGGUGTT‐3′.

### 2.8 Cell proliferation, cycle and apoptosis

Cells were seeded at 1 × 10^4^ cells/well into a Transwell co‐culture system (0.4 μm pore size; Corning Inc, New York, NY, USA) and then treated with various concentrations of the aptamer for 0, 24, 48 and 72 hours. At each time‐point, the number of cells in the upper chamber was counted manually.

Cells were first maintained in medium without serum for 24 hours and then were seeded at 3 × 10^5^ cells/well in a Transwell co‐culture system (0.4 μm pore size) with complete medium containing 4 μM of aptamer for 48 hours. Cells were harvested, washed with PBS, incubated with permeabilization medium (Beckman Coulter) for 1 minute at 37°C and stained with 100 μL of propidium iodide (PI; Beckman Coulter, Beckman Inc, Brea, CA, USA) for 30 minutes at 37°C. The cell cycle distribution was then examined by flow cytometry.

Cells were seeded at 3 × 10^5^ cells/well in a Transwell co‐culture system (0.4 μm pore size) and then were treated with 4 μM of aptamer for 48 hours. After washing with PBS, cells were stained with an Annexin V‐FITC/PI staining kit (BD Biosciences) and analysed by flow cytometry.

### 2.9 Migration assay

As described previously,^23^ 1 × 10^5^ cells/well in 200 μL of medium without serum were seeded in the upper chamber of a Transwell co‐culture system (8 μm pore size; Corning Inc.) with 4 μM of aptamer, and 600 μL of medium with 10% FBS was added to the bottom chamber. After 48 hours incubation, the number of migrated cells present in the bottom chamber was counted.

### 2.10 Adhesion assay

Cells were first labelled with the fluorescent dye Dio (Sigma‐Aldrich, Burlington, VT, USA) for 1 hour at 37°C and were then washed with PBS three times. Cells were co‐cultured directly with HS5 cells in 96‐well plates (5 × 10^4^ cells/well) in the presence of the 4 μM of aptamer for 4 hours at 37°C. Subsequently, the non‐adherent cell fraction was removed by washing with PBS, and the remaining adherent cells were solubilized performed with 1% Triton X‐100 (50 μL/well). Fluorescence intensity at 501 nm was measured performed with a multi‐label plate reader.

### 2.11 Serum stability assay

SL1 (3 μM) was incubated in 100 μL of RPMI 1640 medium with 10% FBS for 1–72 hours at 37°C. At each time‐point, samples were heated to 95°C for enzyme inactivation and then stored at −80°C until all samples were completed. Samples (10 μL each) were mixed with denature gel loading dye and run on 3% agarose gels. The gels were then imaged.

### 2.12 In vivo and ex vivo fluorescence imaging

Four‐week‐old female NCG mice were purchased by biomedical research institute of Nanjing University. Mice were subcutaneously injected with 1 × 10^7^ ARP‐1 cells at the backside. Tumours were allowed to develop until the size reached 0.5‐2 cm in diameter. Mice were anaesthetized performed with a tranquilizer and an anaesthetic, and then 200 μL of physiological saline containing 4.5 nmol of Cy5‐labelled SL1 or a control library was administered systemically via tail vein injection. At certain time‐points, whole body images of live mice were collected by the IVIS Lumina II in vivo imaging system (Caliper Life Science, Shanghai, China).

After in vivo imaging, mice that had been injected with Cy5‐labelled SL1 or a control library were killed by cervical dislocation under narcosis at 1 hour post‐injection. After anatomization, the dissected organs including liver, kidney, spleen, lung, heart and tumour tissue were imaged with the IVIS Lumina II in vivo imaging system. A 640 nm (±15 nm) bandpass filter and a 695‐770 nm bandpass filter were selected as the excitation filter and the emission filter respectively.

### 2.13 Patient sample collections and Isolation of primary CD138+ MM cells

Bone marrow aspirates were collected in EDTA tubes from newly diagnosed myeloma patients. Samples (20 μL) were co‐stained with 250 μM of FAM‐labelled SL1/250 μM of DNA control library and 2 μL of CD138‐APC antibody (BD Biosciences) for 30 minutes at 4°C. The binding ability of SL1 or control library to CD138+ or CD138‐ cells was then analysed by flow cytometry. Primary human CD138+ cells were isolated from BM aspirates performed with a human CD138 enrichment kit (CD138+ Plasma Cell Iso. Kit; Miltenyi Biotec).

### 2.14 Statistical analysis

Data are shown as the mean ± SD for three independent experiments. Statistical significance between groups was analysed by Student's *t* test. One‐way analysis of variance and Fisher's least significant difference test were assessed performed with GraphPad Prism 7.0 (GraphPad software), and the results were used to compare different groups from the GEP dataset. *P* < 0.05 was considered significant.

## 3 RESULTS

### 3.1 C‐met is highly expressed in MM and correlates with poor patient outcomes

**Figure 1:**
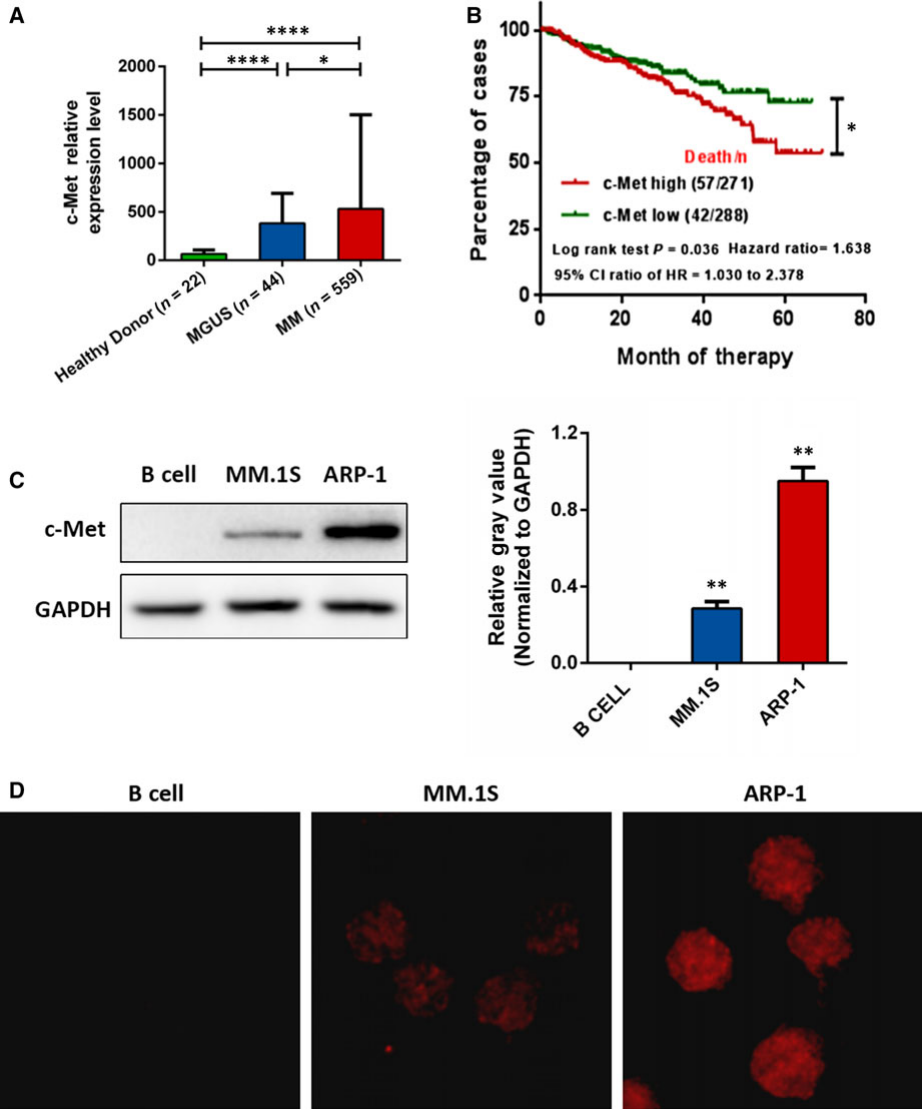
Analysis of c‐met expression in the MM GEP dataset and MM cell lines. A, Relative expression levels of *c‐met* in clinical samples including healthy donors and MGUS and MM patients from the GEP dataset (GSE5900, GSE2658). Data are shown as the mean ± SD, *P < 0.05, ***P < 0.001. B, Univariate survival analysis as determined by the Kaplan‐Meier method. The log‐rank test was used to compare differences between c‐met high and low expressing group in GSE 5900 and GSE 2658, *P* = 0.036, HR = 1.638, 95% CI = 1.030‐2.378. C‐met high and low groups were separated by the median expression. (C) Plasma membrane fractions from B cells, MM.1S cells and ARP‐1 cells were subjected to Western blotting against c‐met. GAPDH was used as the loading control. D, Representative immunofluorescence images showing c‐met (red) localized on the surface membrane of cells

To assess whether c‐met inhibition can be used for MM treatment, we first studied the prevalence of *c‐met* overexpression in MM by analysing the publicly available gene expression profiling (GEP) database of myeloma, GSE 5900 and GSE 2658. In contrast to healthy donors (N = 22), we observed a gradual increase in *c‐met* levels in 44 patients with monoclonal gammopathy of undetermined significance (MGUS) and even higher levels in 559 cases of newly diagnosed MM (Figure 1A). As MM evolves from an asymptomatic pre‐malignant MGUS stage, this result confirms that c‐met is involved in MM disease progression.

We next investigated the prognostic value of *c‐met* levels in MM. The Kaplan‐Meier method was used to estimate overall survival (OS) in the total therapy 2 and 3 (TT2 and 3) cohorts from the GEP dataset. MM patients were separated into *c‐met* high and low expression groups performed with the mean value of gene expression as the cut‐off point. As shown in Figure 1B, OS in MM patients with high *c‐met* expression (*P*_log‐rank_ = 0.036) was significantly reduced with a hazard ratio of 1.638 (95% CI: 1.030‐2.378), suggesting that c‐met expression can serve as a potential prognostic indicator in MM patients. Given that aptamers that are screened via cell‐based SELEX always target proteins on the cell surface, we examined the cellular localization and plasma membrane expression of c‐met in two MM cell lines. Immunofluorescence staining for c‐met in cells revealed that it was exclusively localized on the cell plasma membrane (Figure 1D). Correspondingly, Western blotting analysis for cells membrane extraction showed dramatically increased of c‐met in two MM cell lines in comparison to normal B cells (Figure 1C). Therefore, c‐met can be considered an ideal target for aptamer‐based therapeutics in MM.

### 3.2 SL1 in vitro binding specificity and affinity

**Figure 2:**
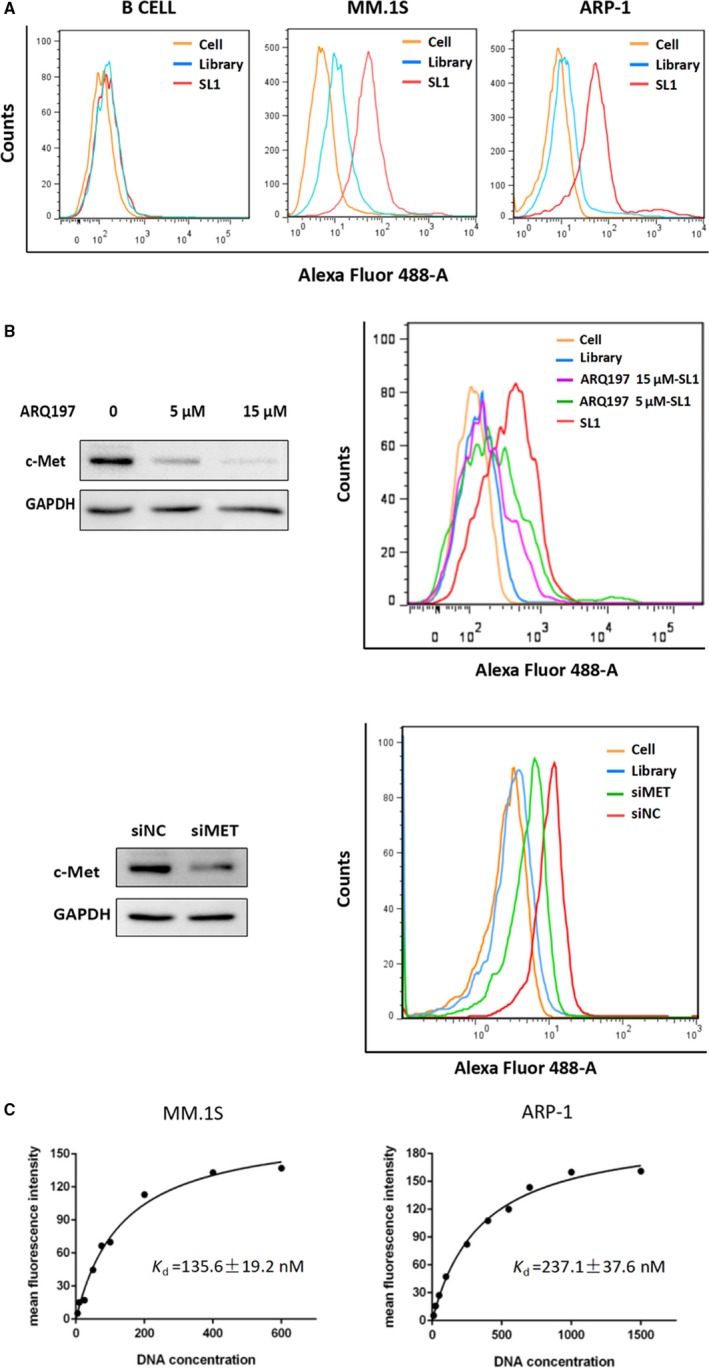
Evaluation of SL1 binding specificity and affinity in vitro. A, Normal B cells, MM.1S cells and ARP‐1 cells were incubated separately with either FAM‐labelled SL1 (red lines) or a FAM‐labelled random DNA control library (blue lines) and then analysed by flow cytometry. Unstained cells served as the negative control. For simplicity, only the saturated concentration of SL1 and an equal molar amount of the control library are shown. B, Western blotting analysis confirmed that cell surface expression of c‐met was significantly decreased by treatment with 5 or 15 μM of the c‐met inhibitor ARQ197 and 100 nM of the c‐met siRNA in MM.1S cells. GAPDH was used as the loading control. Accordingly, flow cytometry showed that the binding ability of SL1 was dramatically reduced in groups treated with ARQ197 plus SL1‐treated groups (purple and green lines) or the siRNA‐c‐met group (green line), compared to the group treated with SL1 alone (red line) or siRNA control group (red line) respectively. C, The dissociation constant of SL1 for MM cells. Moreover, 3 × 10^5^ cells were incubated with different concentrations of FAM‐labelled SL1. The actual fluorescence intensity that the mean fluorescence intensity of the DNA library at each concentration was subtracted from and the mean fluorescence intensity of SL1 were fitted into SigmaPlot to determine the *K*_*d*_ value

To evaluate the binding specificity of SL1 for native cellular c‐met, c‐met‐positive MM cell lines, MM.1S and ARP‐1, and c‐met‐negative cell lines were incubated with different concentrations of FAM‐labelled SL1 or a random DNA control library. Cellular fluorescence intensity was detected by flow cytometry. The results show that compared to the control library and unstained cells, a significant increase in fluorescence signal was observed in the two MM cells but not in normal B cells (Figure 2A). In contrast, knockdown of c‐met expression by the c‐met inhibitor ARQ197 or c‐met specific siRNA reduced the fluorescence signal/shift (Figure 2B), indicating that SL1 selectively bound c‐met protein expressed in MM cells, thereby differentiating MM cells from normal cells.

The equilibrium dissociation constant (*K*_*d*_) was used to quantitatively evaluate the c‐met binding affinity of SL1. MM.1S or ARP‐1 cells were incubated with increasing concentrations of FAM‐labelled SL1, and the cells were analysed by flow cytometry. *K*_*d*_ values were determined by fitting the dependence of fluorescence intensity of the cell/aptamer complex to aptamer concentration with the equation *Y* = *B* max *X*/(*K*_*d*_ + *X*). From this equation, the *K*_*d*_ of SL1 was estimated to be 135.6 nM for MM.1S and 237.1 nM for ARP‐1 cells (Figure 2C). As SL1 showed nanomolar binding affinity for both MM cell lines that is comparable to the binding affinity of antibodies, these data support the use of SL1 as a potential new therapeutic strategy for treating MM.

### 3.3 Fluorescence imaging of SL1 in vivo and ex vivo

**Figure 3:**
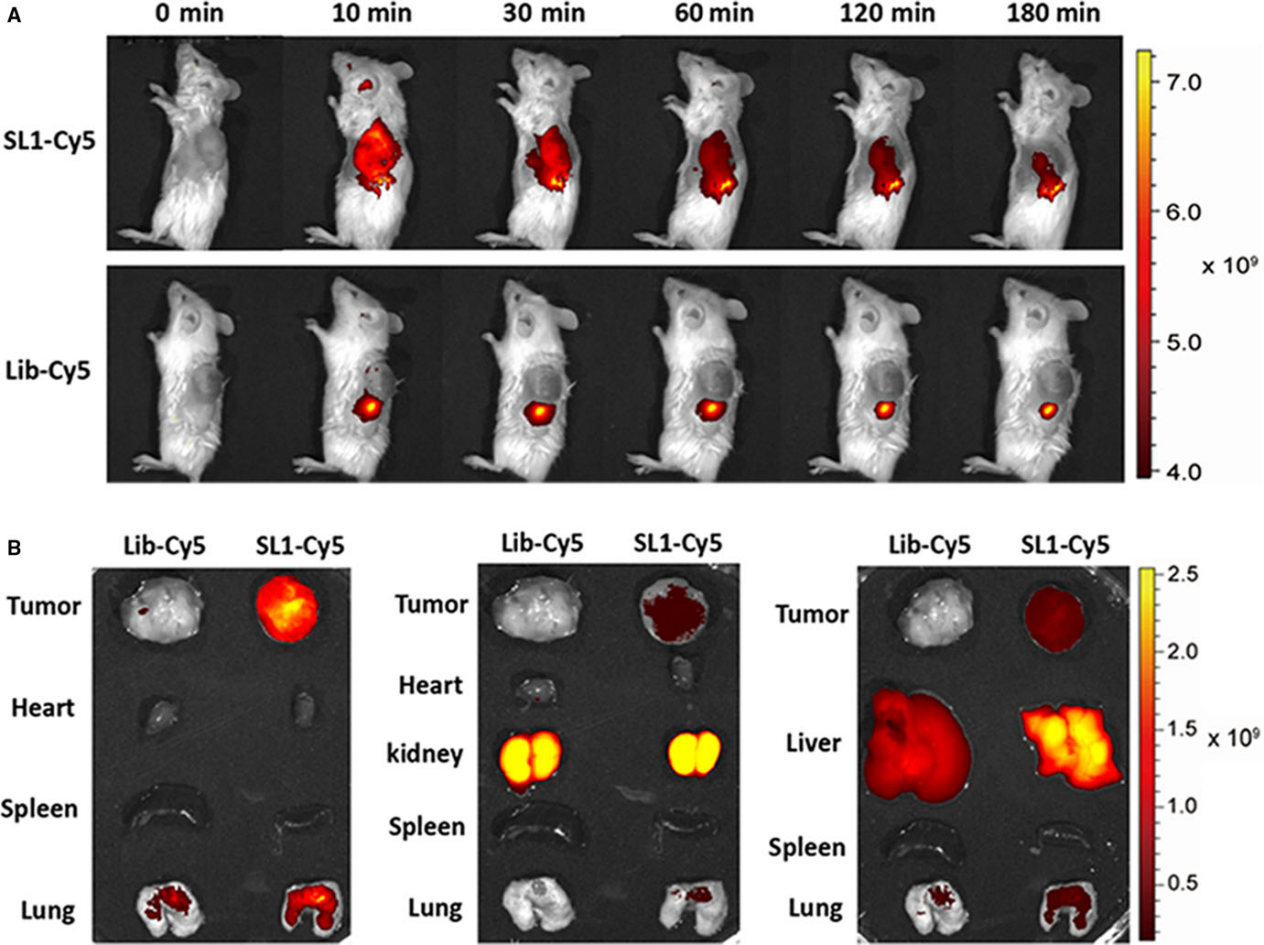
*In vivo* and ex vivo biodistribution of SL1 in a xenograft mouse model of MM. A, Upper: time‐lapse in vivo fluorescence imaging after injection of Cy5‐labelled SL1. Lower: time‐lapse in vivo fluorescence imaging after injection of a Cy5‐labelled library. Representative images are shown (mice, n = 3). B, Ex vivo fluorescence imaging of dissected organs and tumour tissues at 1 hour after injection of Cy5‐labelled SL1 or a Cy‐5‐labelled control library. Representative images are shown (mice, n = 3)

To study whether SL1 retained binding specificity in vivo, Cy5‐labelled SL1 or a Cy5‐labelled control library was intravenously injected into ARP‐1‐bearing NCG mice for in vivo fluorescence imaging. After injection, the spatial and temporal biodistribution of the aptamer was determined at 10, 30, 60, 120 and 180 minutes. The results show that high levels of fluorescent signal could be observed in tumour sites from 10 to 60 minutes post injection, followed by a gradual decrease in signal thereafter. However, the mice injected with Cy5‐labelled SL1 showed higher fluorescent signal than mice injected with the Cy5‐labelled control library, indicating that SL1 effectively targets MM tumours in vivo with high selectivity and sensitivity (Figure 3A).

Whole body distribution of SL1 was assessed via ex vivo fluorescence imaging. Dissected tumour tissues and organs including heart, lung, spleen, liver and kidney were obtained 60 minutes after injecting Cy5‐labelled SL1 or the Cy5‐labelled control library. Imaging revealed high SL1 accumulation in tumour sites. A fluorescent signal was also observed in kidney tissue, which suggests that SL1 is excreted and cleared by the kidneys (Figure 3B). These results suggest that SL1 targets c‐met expressing tumours in vivo and that SL1 could serve as a potential molecular probe for MM diagnosis and therapy.

### 3.4 SL1 inhibits MM cell growth in vitro in an HS5 co‐culture model

**Figure 4:**
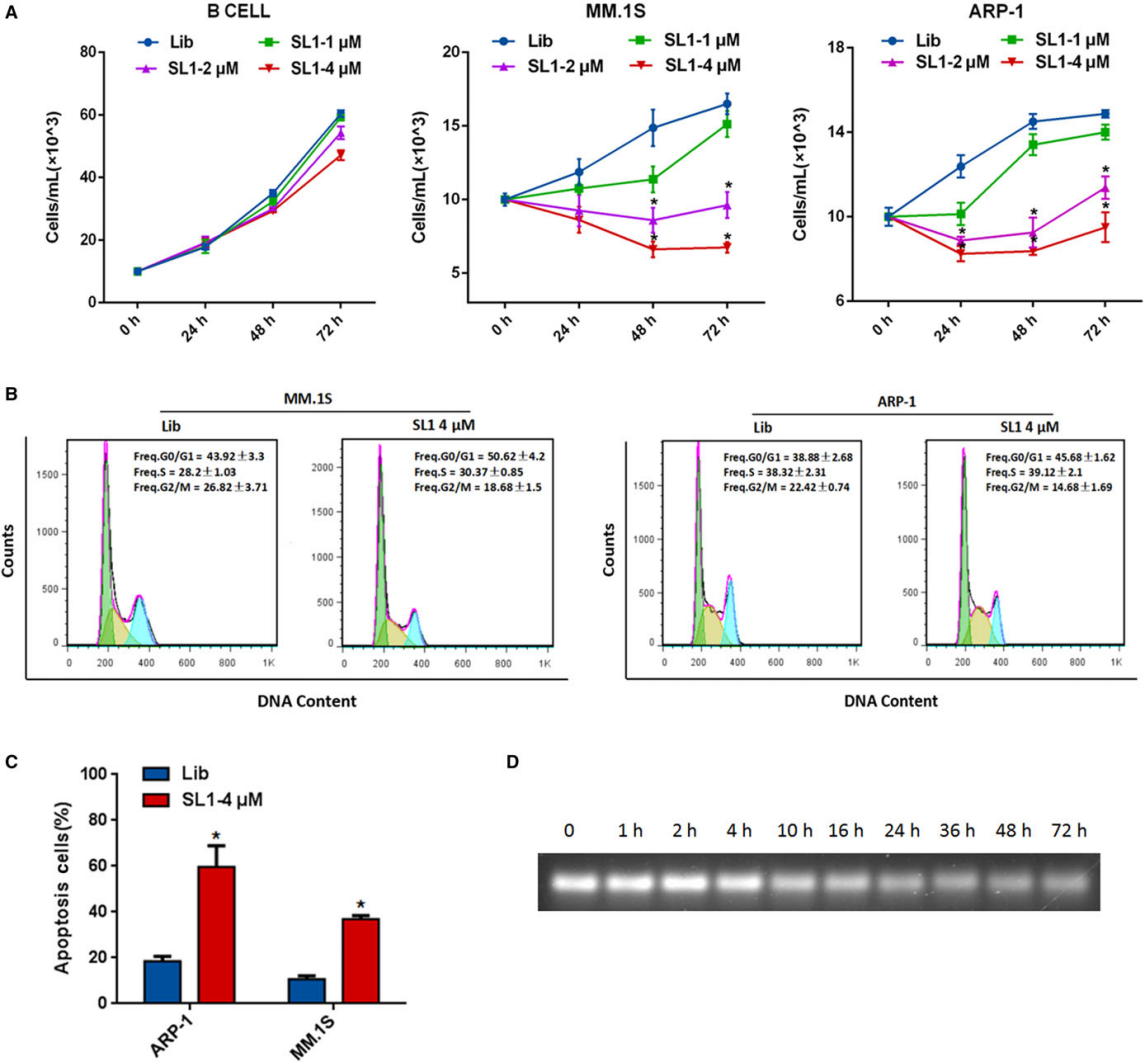
Effects of SL1 on MM cell proliferation, cell cycle and apoptosis in an HS5 co‐culture model. Cells were plated in the upper chamber of a Transwell co‐culture system with the specific concentration of SL1 indicated or a DNA control library (4 μM). A, The co‐culture continued for 24, 48 or 72 hours. Cells were harvested and assessed for cell viability by manual cell counting. Data are presented from three independent experiments as the mean ± SD, *P < 0.05. B, Cells were synchronized at G0/G1 phase by serum starvation for 24 hours. After co‐culturing with HS5 cells for 48 hours, cells were harvested, stained with PI, and cell populations were sorted by flow cytometry into G0/G1, S and G2/M phases. Data are shown as the mean ± SD,* P* < 0.05. C, After co‐culturing with HS5 cells for 48 hours, cells were harvested, stained with annexing V‐FITC/PI, and the percentage of apoptotic cells was determined by flow cytometry. Data are shown as the mean ± SD from three independent experiments, *P < 0.05. D, Serum stability assays were performed to assess the stability of SL1 in cell medium containing 10% FBS. While SL1 gradually degraded after 1 hour, but it was still detected after 72 hours in 10% FBS

In addition to binding c‐met, we further explored the therapeutic potential of SL1 in MM performed with an in vitro co‐culture model. Given the supportive role of BMMEs in disease progression in malignant PC, a co‐culture system performed with the human BM stromal cell line HS5 was explored. Co‐culture with HS5 stromal cells was used to model HGF production by the BMME in vitro. First, the proliferation of B cells, MM1.S cells and ARP‐1 cells in the presence of SL1 and HS5 stromal cells was evaluated. As shown in Figure 4A, SL1 inhibited the proliferation of two MM cell lines in a dose‐dependent manner, particularly at 2 and 4 μM but had almost no effect on B cells. Importantly, the inhibition of cell proliferation by SL1 correlated with c‐met expression. Time‐dependent inhibition of proliferation was not observed at any given dose of SL1, perhaps due to poor stability/rapid degradation of the oligonucleotide aptamer under the in vitro culture conditions (Figure 4D). Nevertheless, SL1 clearly showed anti‐proliferative effects in MM cells but not in B cells.

To further understand the anti‐proliferative effects of SL1, we investigated the impact of SL1 (4 μM) on cell cycle and apoptosis at 48 hours via PI‐staining and annexin V‐FITC/PI staining assays respectively. As shown in Figure 4B, after serum deprivation for 24 hours to allow for cell cycle synchronization and then re‐feeding with 10% serum in a co‐culture system for 48 hours, SL1 treated MM.1S or ARP‐1 cells showed a decrease in the G2/M phase population compared to library‐treated control cells. Accordingly, SL1‐treated cells showed a significant increase in the percentage of cells in the G0/G1 phase. Moreover, cell apoptosis was dramatically increased in SL1‐treated cells compared to library‐treated control cells (Figure 4C). These data indicate that inhibition of cell cycle progression and induction of cell death both contribute to the observed SL1‐mediated anti‐proliferative effect.

### 3.5 SL1 prevents cell migration and adhesion in an HS5 co‐culture model, and suppresses HGF‐induced activation of c‐met signaling in vitro

**Figure 5:**
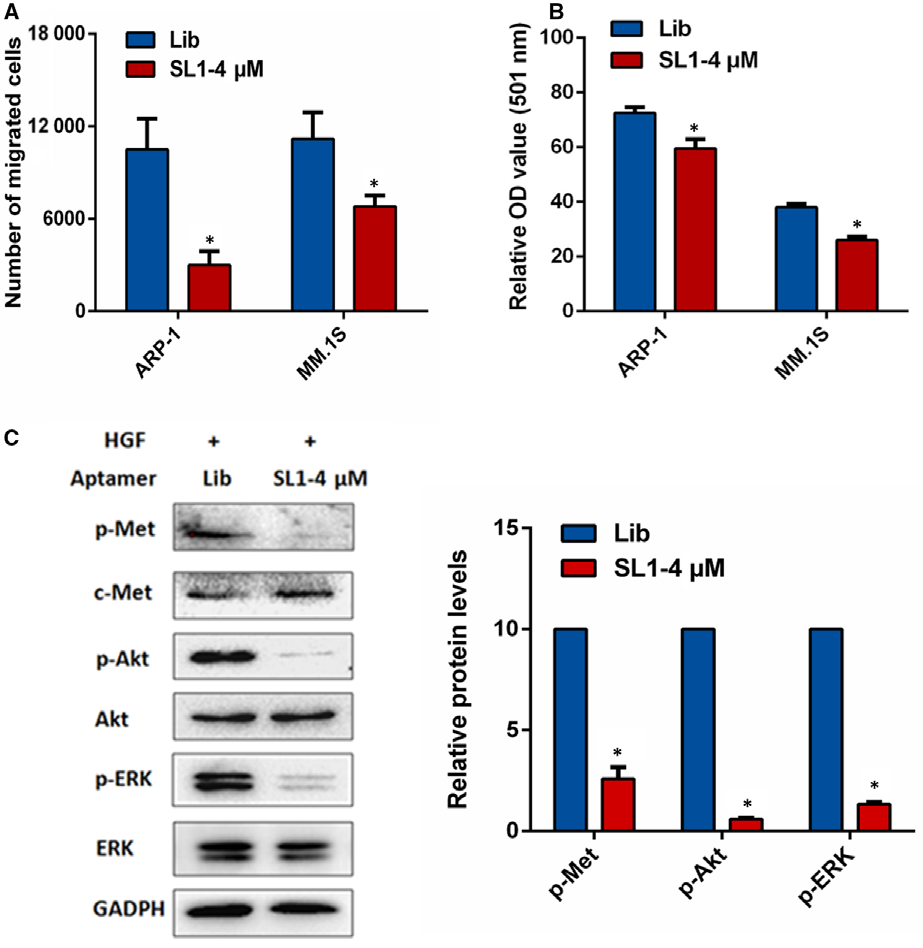
Effects of SL1 on MM cell migration and adhesion in an HS5 co‐culture model and HGF‐induced c‐met signaling. A, HS5 cells were pre‐seeded in the bottom wells of Transwell migration chambers for 24 hours. Then, MM1.S or ARP‐1 cells were seeded in the top wells of chambers containing serum‐free media with 4 μM of SL1 or 4 μM of library control. After 48 hours, cell migration was measured as described in the Section 2. Data are means ± SD from three independent experiments, *P < 0.05. B, Adhesion of MM1.S or ARP‐1 cells to HS5 monolayers was evaluated by a cell adhesion assay as described in the Section 2. Data are means ± SD from three independent experiments, *P < 0.05. C, ARP‐1 cells were pre‐stimulated with HGF (110 pmol/L) for 20 minutes before the addition of 4 μM of SL1 or 4 μM of library control for 48 hours. After 48 hours, expression analysis was performed for p‐c‐met/c‐met, p‐AKT/AKT and p‐ERK/ERK by Western blotting. GAPDH served as the loading control. Right panel: densitometric analysis and statistical analysis (n = 3 independent experiments) of protein bands performed with ImageJ software. *P < 0.05 versus the loading control

Multiple myeloma has a disseminated growth pattern throughout the BM and is dependent on the migration of cells across endothelial barriers and on adhesion to other cells and BMME components.^24^ As HGF/c‐met signaling elicits MM cell migration and adhesion, we investigated the effects of SL1 on MM cell migration and adhesion via Transwell migration and cell adhesion assays in a co‐culture system. As shown in Figure 5A, HS5 cells were added to the bottom compartment of transwell chambers to give a confluent monolayer. The migration of cells across an 8 μm pore filter was significantly inhibited in the presence of SL1 relative to library treated control cells. In addition, SL1 treatment significantly prevented adhesion of MM.1S or ARP‐1 cells to a confluent monolayer of HS5 cells (Figure 5B).

It is known that HGF/c‐met signaling promotes the adhesion and migration of myeloma cells by stimulating multiple downstream pathways including PI3K/AKT and Ras/ERK.^25^ To confirm that SL1‐mediated effects are a result of HGF/c‐met pathway inhibition, we investigated the effects of SL1 on the expression levels of p‐c‐met (Y1349), p‐ERK and p‐AKT after stimulating with HGF. The results show that HGF‐stimulated phosphorylation of c‐met, ERK and AKT was dramatically reduced in the presence of SL1, while their total protein levels remained unchanged (Figure 5C). Therefore, SL1 blocks HGF/c‐met signaling and the phenotypic effects that typically result from HGF/c‐met pathway stimulation.

### 3.6 Aptamer SL1 specifically recognizes and suppresses primary CD138 PC from MM patients

**Figure 6:**
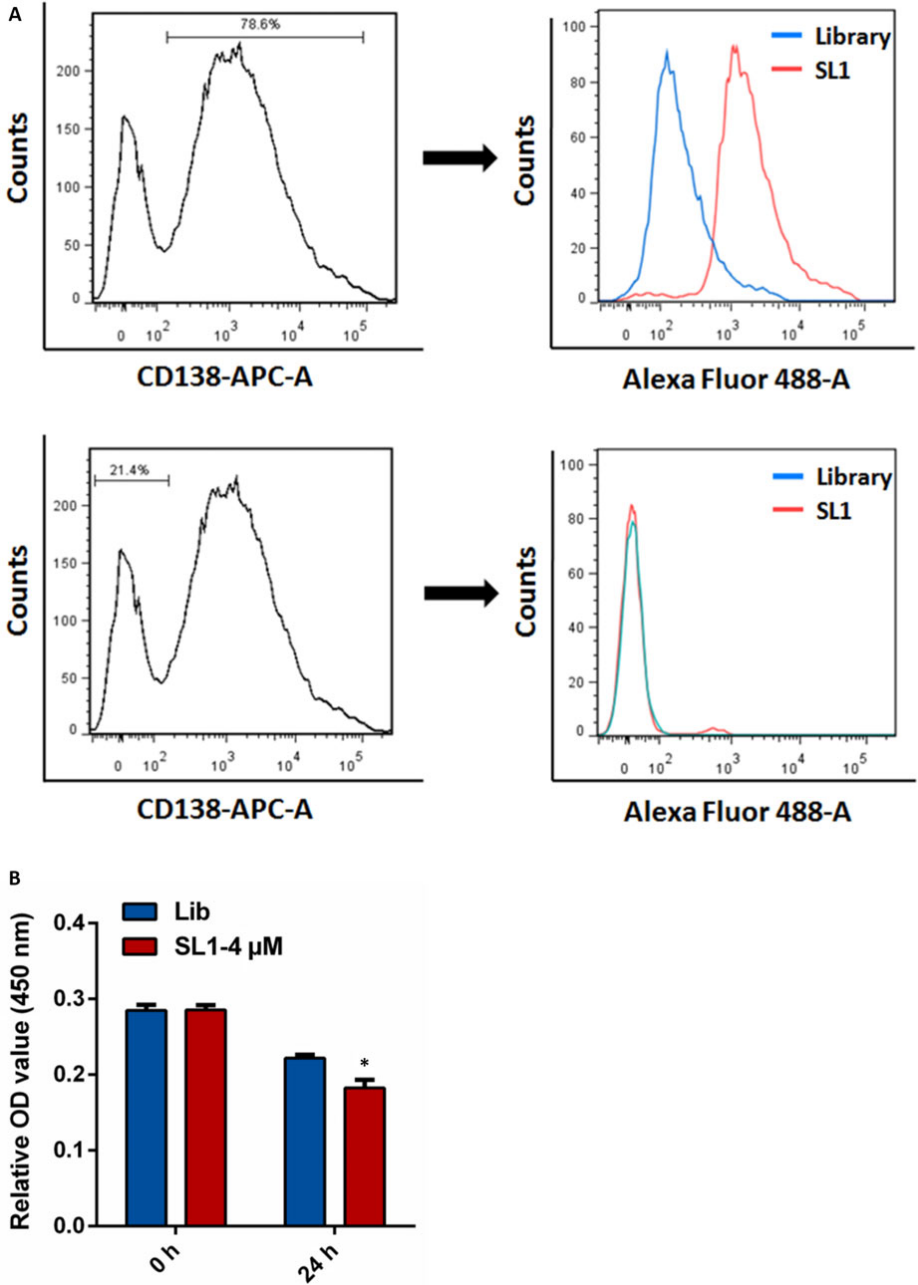
SL1 binding to human MM CD138‐positive bone marrow cells. A, Left: Analysis of the ratio of CD138+ and CD138‐ cells in BM from a representative MM patient performed with flow cytometry. CD138+ cells accounted for 78.6% of the cell population, and the remaining cells were CD138‐; Right: CD138+ or CD138‐ MM cells were incubated separately with either 250 μM FAM‐labelled SL1 (red lines) or 250 μM of a FAM‐labelled control library (blue lines) and analysed by flow cytometry. DNA library groups served as the negative control. B, Proliferation of isolated CD138+ cells after treatment with 4 μM SL1 or 4 μM of a control library was evaluated by a CCK‐8 assay. The results are the mean ± SD of four independent experiments, *P < 0.05

CD138 is a well‐known surface antigen for MM and PC in BM.^26^ To determine whether SL1 can recognize MM cells from clinical specimens, BM samples from patients (n = 4) were stained with CD138‐APC antibody and co‐stained with FAM‐labelled SL1 or the DNA control library. The results show that SL1 specifically binds CD138+ cells but not CD138‐ cells (Figure 6A).

We isolated primary CD138+ cells from the BM of MM patients (n = 3) performed with CD138 positive selection kits and examined their growth upon treatment with 4 μM of SL1 or 4 μM of the library control. Proliferation of CD138+ cells was significantly inhibited by SL1 compared to cells treated with the DNA control library (Figure 6B). These findings suggest that the inhibitory effects of SL1 are observed not only in in vitro cell line systems but also in clinical MM cells.

### 3.7 SL1 synergizes with BTZ in vitro to inhibit MM

**Figure 7:**
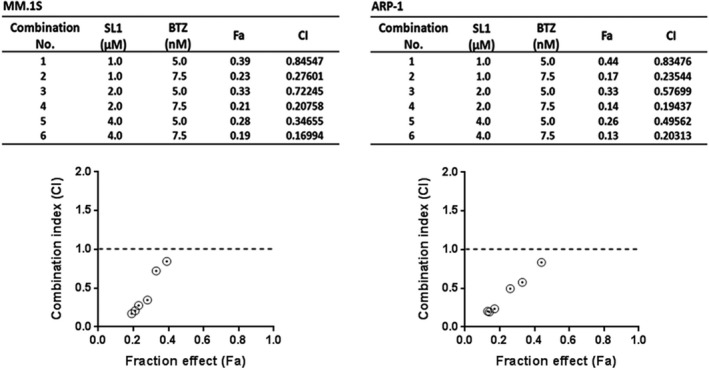
Evaluation of the synergistic effects of SL1 and BTZ in MM cells. A, MM.1S and ARP‐1 cells were treated with SL1 alone, BTZ alone or a combination of SL1 plus BTZ as indicated for 24 hours and were then assessed for cell viability performed with a CCK‐8 assay. Combination index (Cl) corresponding to the specific data points on the table were analysed by the CompuSyn software for non‐constant drug ratio and plotted on the graph against fraction effect (Fa) to evaluate SL1‐BTZ synergy. A Cl <1.0 indicates synergism. 0.1‐0.3, strong synergism; 0.3‐0.7, synergism; 0.7‐0.85, moderate synergism; 0.85‐0.9, slight synergism

Bortezomib is a first‐line treatment for MM. c‐met signaling is frequently activated in relapsed and resistant MM, where it plays an important role in induction of BTZ resistance.^27^ To assess the feasibility of combining SL1 and BTZ, MM.1S and ARP‐1 cells were treated with different concentrations of SL1 alone, BTZ alone or a combination of SL1 and BTZ for 24 hours. Dose selection was based on the IC50 values for SL1 and BTZ. The degree of synergism between SL1 and BTZ was determined performed with CompuSyn Software. Fa‐Cl curves showed that a combination of SL1 and BTZ synergistically reduced cell growth in both MM cell lines (Figure 7A).

## 4 DISCUSSION

The development of MM depends not only on oncogenic events occurring in MM cells but also on the extracellular BMME, which plays a key role in MM cell growth, survival, homing and drug resistance. “Normalizing” the tumour microenvironment or inhibiting communication between MM cells and their surrounding microenvironment is an important therapeutic strategy.^28^ HGF/c‐met signaling is a key pathogenic factor in the BMME. HGF is frequently present in the BMME where it activates the c‐met receptor in MM cells in a paracrine or autocrine fashion.^5^ We evaluated the mRNA expression of c‐met in two independent publicly available data sets and showed that c‐met transcript levels gradually increased from healthy donors with low expression to MGUS patients with intermediate expression and then to MM patients with high expression. Kaplan–Meier survival analysis shows that patients with higher expression of c‐met have significantly shorter OS. These observations indicate that c‐met is involved in the progression of MM and represents an independent factor associated with poor prognosis. Aberrant c‐met activation can also occur through HGF‐independent mechanisms such as *c‐met* mutations or gene amplification. We found that c‐met is significantly overexpressed on the cell surface of MM cells compared to normal B cells, supporting the use of c‐met as a therapeutic biomarker for MM.

While multiple antibodies that target c‐met or HGF are under preclinical and clinical development, none of them have shown significant clinical benefit to patients.^29^ Their potential immunogenicity and high production costs are of particular concern for this modality. In addition, selective c‐met small‐molecule inhibitors have failed to gain approval due to adverse effects, dose‐limiting toxicities or acquired resistance; therefore, novel approaches that suppress HGF/c‐met signaling are needed.^29^ In the last decade, aptamers have emerged as attractive alternatives to antibodies and small molecules and have found use in diagnostic, therapeutic, imaging and targeting applications.^30^ A handful of aptamers have been developed to function as therapeutic agonists in cancer. For example, DNA aptamers targeting nucleolin, PDGF, and HER2 and RNA aptamers targeting VEGF, HER3, and EGFR have been explored.^16^ In myeloma, two aptamers have been developed as antagonists to inhibit the interaction between protein with its ligand. NOX‐A12, an RNA oligonucleotide with L‐configuration that binds and neutralizes the C‐X‐C motif chemokine ligand 12 (CXCL12), is one such aptamer. NOX‐A12 has been shown to decrease tumour metastasis and drug resistance caused by cancer cell homing.^31^ A CD38‐specific ssDNA aptamer drug conjugate (ApDC) targets drug delivery to CD38‐expressing MM cells and releases the drug payload intracellularly.^18^ However, the use of this ApDC to target c‐met in MM has yet to be reported. The aptamer CLN3 is the only known c‐met‐binding DNA aptamer. The minimal binding domain of CLN3, SL1, was found to retain high binding affinity for c‐met and blocked the HGF/c‐met interaction and c‐met signaling in SNU‐5 cells.^21^ In this study, we demonstrate that SL1 has high specificity and affinity to membrane‐bound c‐met in MM. In vitro, SL1 selectively bound to c‐met‐positive MM cells with a *K*_*d*_ of nanomolar level but not to c‐met‐negative B cells. Down‐regulation of c‐met abrogated the binding of SL1 to MM cells. In vivo, injection of fluorescently labelled SL1 in mice that harboured c‐met‐positive MM cells resulted in SL1 enrichment in tumour areas. These results suggest that SL1 could potentially be used to target c‐met‐positive MM cells, for example, as part of an aptamer‐based drug delivery system or in imaging studies.

In addition to binding cellular c‐met, we found that SL1 possesses tumour inhibitory activity. Ueki et al reported that SL1 inhibits metastasis‐related behaviour, such as the scattering and migration of SNU‐5 cells.^21^ Similarly, Piater et al described a truncated version of CLN3 (CLN3‐T) that suppressed HGF‐induced migration of NCI‐H441 cells and invasion of MDA‐MB‐231 cells and inhibited c‐met phosphorylation.^32^ In our study, we showed that SL1 markedly inhibited the growth of c‐met‐positive MM cells, while it had no effect on B‐cells. SL1 suppressed HGF‐induced activation of c‐met and downstream signaling by ERK and AKT, resulting in cell cycle arrest, cell apoptosis, inhibition of cell migration and reduced cell adhesion in MM cells. In addition, SL1 was stable in 10% FBS over 72 hours, which suggests that SL1 may be suitable for in vivo applications.

Importantly, we found that SL1 has a much greater affinity for CD138+ cells than for CD138‐ cells isolated from the BM of MM patients. Furthermore, SL1 showed antiproliferative effects on primary CD138+ cells. CD138 is a specific surface marker of MM PC. As a co‐receptor, CD138 strongly promotes HGF‐induced signaling by promoting the dimerization of c‐met.^33^ c*‐met* mRNA and protein expression were found to be higher in CD138+ cells than in CD138‐ cells.^11^ These results support the potential clinical use of SL1 by only targeting MM cells as opposed to healthy cells.

SL1 and BTZ were found to act synergistically in vitro as a combination of SL1 and BTZ gave enhanced inhibition of MM cell proliferation compared to either compound alone. In the clinic, c‐met expression levels can affect a patient's response to BTZ. For example, higher c‐met levels are associated with poor response and outcome in myeloma patients treated with BTZ‐based therapies.^11^ Knockdown of c‐met by shRNA in vitro increases sensitivity to BTZ in MM U266 cells.^34^ Furthermore, SU11274, a novel selective c‐met inhibitor, is known to induce apoptosis and necrosis, and it can reverse BTZ resistance in R5 cells.^27^ Our results suggest that SL1 is comparable to other BTZ‐based combination treatments for MM. SL1 in combination with BTZ significantly increased BTZ's therapeutic activity against MM and has the potential to improve patient survival.

In conclusion, SL1 is the first c‐met‐specific DNA aptamer with potential application in targeting and treating MM. SL1 can serve not only as a molecular probe to selectively recognize cellular c‐met with high affinity in vitro and in vivo, but it can also serve as a therapeutic antagonist for c‐met positive MM cell lines and primary CD138+ MM cells from clinical samples through the inhibition of HGF‐induced c‐met signaling. Furthermore, in combination with SL1, BTZ exhibits increased cytotoxicity. Given that no other aptamer has been explored as a c‐met‐targeting antagonist in MM, our data warrant further clinical development of this novel therapeutic aptamer of c‐met.

## CONFLICT OF INTEREST

The authors confirm that there are no conflicts of interest.

## AUTHOR CONTRIBUTION

YZ, WZ, SS, HZ and YZ performed research and analysed the data; JL, JZ, YY, LL, XX, MY and ZW designed experiments; HG and JS collected patient's samples; YZ, ZW and JL wrote and edited the paper.
